# Institutional and matrix support and its relationship with primary healthcare

**DOI:** 10.1590/S0034-8910.2015049005519

**Published:** 2015-09-10

**Authors:** Alaneir de Fátima dos Santos, Antônio Thomaz Gonzaga da Matta Machado, Clarice Magalhães Rodrigues dos Reis, Daisy Maria Xavier Abreu, Lucas Henrique Lobato de Araújo, Simone Cristina Rodrigues, Ângela Maria de Lourdes Dayrell de Lima, Alzira de Oliveira Jorge, Délcio Fonseca

**Affiliations:** Núcleo de Educação em Saúde Coletiva. Faculdade de Medicina. Universidade Federal de Minas Gerais. Belo Horizonte, MG, Brasil

**Keywords:** Primary Health Care, organization & administration, Health Planning Support, Cross-Sectional Studies

## Abstract

**OBJECTIVE:**

To analyze whether the level of institutional and matrix support is associated with better certification of primary healthcare teams.

**METHODS:**

In this cross-sectional study, we evaluated two kinds of primary healthcare support – 14,489 teams received institutional support and 14,306 teams received matrix support. Logistic regression models were applied. In the institutional support model, the independent variable was “level of support” (as calculated by the sum of supporting activities for both modalities). In the matrix support model, in turn, the independent variables were the supporting activities. The multivariate analysis has considered variables with p < 0.20. The model was adjusted by the Hosmer-Lemeshow test.

**RESULTS:**

The teams had institutional and matrix supporting activities (84.0% and 85.0%), respectively, with 55.0% of them performing between six and eight activities. For the institutional support, we have observed 1.96 and 3.77 chances for teams who had medium and high levels of support to have very good or good certification, respectively. For the matrix support, the chances of their having very good or good certification were 1.79 and 3.29, respectively. Regarding to the association between institutional support activities and the certification, the very good or good certification was positively associated with self-assessment (OR = 1.95), permanent education (OR = 1.43), shared evaluation (OR = 1.40), and supervision and evaluation of indicators (OR = 1.37). In regards to the matrix support, the very good or good certification was positively associated with permanent education (OR = 1.50), interventions in the territory (OR = 1.30), and discussion in the work processes (OR = 1.23).

**CONCLUSIONS:**

In Brazil, supporting activities are being incorporated in primary healthcare, and there is an association between the level of support, both matrix and institutional, and the certification result.

## INTRODUCTION

In Brazil, the construction of a model that places primary care as a priority among the healthcare services imposes challenges that are handled with proposals aiming to implement comprehensive care in the Brazilian Unified Health Care System (SUS). To debate the traditional ways through which health care services are provided – they maintain fragmented work processes that are centered on procedures, operating under a hegemonic biomedical model – proposals are made by SUS concerning institutional support (IS) and matrix support (MS).^2^

IS is structured under a co-management model and it aims to develop and enhance supervision and assessment strategies for health care services.^a^ Such proposal implies a collected negotiation space between management and care, fostering the participation of individuals in organized collective groups aiming to produce goods or services, and to promote permanent education and the shared management of the institution and its work processes.^5^

The systematic production of official documents that deal with IS strategy started in 2003, and it was influenced by *Apoio Paideia*, developed by Campos.^5^ Under that concept, management and health care practices get closer to each other in the attempt to develop democratic management – co-management.^b^ ^,^ ^c^

MS, in turn, is a work organization strategy in which health care professionals must work in multi-professional teams.^2^ Thus, the support from qualified professionals to primary healthcare (PHC) teams may ensure full care, proposing mutual responsibility with the users of the service.^2^ MS invests in management and care structure changes for health care services, in a way to create an organization which fosters the commitment from the teams to the production of health, without demanding omniscience or omnipotence from them, and allowing them to feel personally and professionally fulfilled. MS thus refers to a new arrangement that fosters the production of new inter-relationship patterns between teams and users and which gets professionals more committed to the production of health, overcoming organizational and communication obstacles.^4^

The MS device may bring problems into the practices and may show paths for the construction of another care model.^12^ The first MS experiences first took place in 1989 in Campinas, SP, Southeastern Brazil, in two mental health care services that started operating with multi-professional teams. It was later expanded in Betim, MG, Southeastern Brazil, and, in 2003, the Ministry of Health adopted it in the structuring of mental health care.^4^ In 2009, the Ministry of Health implemented MS with the institutionalization of *Núcleos de Apoio à Saúde da Família* (Family Health Care Support Centers) in the primary healthcare organizational structure at SUS. Some studies show the benefits from MS, especially in mental health care.^9^ ^,^ ^10^ ^,^ ^12^ ^,^ ^13^ ^,^ ^15^

Considering that those mechanisms are expected to advance, it is necessary to characterize the situation of IS and MS in BHC. To do so, the external assessment of *Programa de Melhoria do Acesso e Qualidade da Atenção Básica *(PMAQ-AB – Program for the Improvement of Primary Healthcare Access and Quality)^d^ provides information on BHC work processes, considering country-wide IS and AM dimensions.

This study aimed to analyze whether the level of institutional and matrix support is associated with better certification of primary healthcare teams.

## METHODS

This is a cross-sectional study based on the first cycle of PMAQ-AB’s external assessment database. Interviews were conducted with 17,055 university professionals from the BHC team in 2012, and they were distributed in all Brazilian states. The data regarding the teams that received IS and MS activities were analyzed and corresponded to 14,306 (84.0%) and 14,489 (85.0%), respectively.

We used the variables from questionnaire II of PMAQ-AB, listing IS and MS activities that are provided to evaluated teams. The IS and MS types that are considered by PMAQ-AB are shown in detail in Table 1. We also considered the result that was obtained by the teams in the certification that was adopted by PMAQ-AB: very good, good, and fair.

**Table 1:** Types of institutional and matrix support that were analyzed by *Programa Nacional de Melhoria do Acesso e da Qualidade da Atenção Básica* (PMAQ-AB – Program for the Improvement of Primary Healthcare Access and Quality). Brazil, 2012.

Institutional support	n	%	Matrix support	n	%
Discussion on work processes from primary healthcare teams	12,893	90.1	Clinical appointments	12,825	88.5
Support to self-assessment	11,558	80.8	Discussion on clinical cases	10,662	73.6
Supervision and evaluation of indicators	11,773	82.3	Shared clinical actions	10,547	72.8
Team planning and organization	4,723	33.0	Joint construction of therapeutic processes	7,921	54.7
Shared evaluation	9,760	68.4	Permanent education	8,936	61.7
Workshop with a specific objective	6,955	48.6	Discussions on work processes with teams	3,308	22.8
Permanent education	9,068	63.4	Interventions in the territory with teams	9,400	64.9
Meetings with teams	10,510	73.5	Visits with professionals	10,507	72.5
Other kinds of support	441	2.5			
Total	14,306			14,489	

Source: Ministry of Health.^d^

Initially, the supporting activities that were received by teams were verified through absolute and relative frequencies. The level of support variable was created, concerning the extent of support that was received by the teams. That variable was calculated through the sum of MS and IS activities that were received by each team. Then, the IS and MS levels were classified according to the number of supporting activities received, as follows: low support (one to three activities), medium support (four to six activities), and high support (seven to eight activities).

To check for the associations between the support received by the teams and the quality of care, a binary multiple logistic analysis was conducted, and its dependent variable was the result from the certification of teams. That variable was classified as “very good or good” or “fair or insufficient” certification, and two multiple logistic regression models were applied. In the first model, the independent variable was the level of support from the teams for IS and MS, and “IS and MS activities that were identified in PMAQ-AB” was the independent variable in the second one. The multivariate analysis considered the variables with p < 0.20 in the univariate analysis. A statistic significance level of p < 0.05 and a confidence interval of 95% (95%CI) were adopted, and respective odds ratios (OR) were calculated to indicate the extent of associations. OR and 95%CI estimates for each variable were adjusted for the effect of the remaining studied variables in the model. The final multiple model adjustment was conducted with Hosmer-Lemeshow test.

## RESULTS

Table 1 shows the detailed relative and absolute frequencies of the institutional and matrix support types that were analyzed by PMAQ-AB. The main IS activities were: discussion on the work processes of primary healthcare teams (90.1%), supervision and assessment of indicators (82.3%), and support to self-assessment (80.8%); team planning and organization had the lowest frequency (33.0%) (Table 1). Concerning MS, the activities that were the most present in BHC teams were: clinical appointments (88.5%), discussion on clinical cases (73.6%), shared clinical actions (72.8%), and visits with professionals (72.5%); discussion on work processes with teams was found to be the MS activity that involved less teams (22.8%).

We observed that most teams had more than one type of support. Only 3.6% and 8.8% of them received a single IS or MS activity, respectively. In regards to IS, 17.1% of Family Health Teams had low support, 47.7% of them had medium support, and 34.8% of them had high level of support. In regards to MS, 25.9% of teams had low support; 38.7% and 35.5% of them had medium and had high levels of support, respectively (Table 2).

**Table 2:** Distribution of percentages for institutional and matrix support types (PMAQ-AB). Brazil, 2012.

Number of types of support	Level of support	Institutional support		Matrix support	

		n	%	n	%
0		62	0.4	–	–
1	Low	522	3.6	1,271	8.8
2		758	5.3	1,113	7.7
3		1,179	8.2	1,362	9.4
4	Medium	1,585	11.1	1,483	10.2
5		2,298	16.1	1,853	12.8
6		2,932	20.5	2,272	15.7
7	High	3,487	24.4	3,386	23.4
8		1,483	10.4	1,749	12.1
Total		14,306	100	14,489	100

Source: Ministry of Health.^d^

To analyze the association between the level of support and certification, the dependent variable reference category was considered to be the fair certification and “very good or good” the answer. Increased chances of having very good or good certification were observed, according to the support level received. For IS, we observed 1.96 and 3.77 chances for teams who had medium and high levels of support to have very good or good certification ratings, respectively. For AM, the chances of their having very good or good certification were 1.79 and 3.29, respectively. That is, receiving high level of support, whether it is matrix or institutional, increases the chances for teams to receive better certification ratings (Table 3).

**Table 3:** Association among institutional and matrix support levels and team certification (PMAQ-AB). Brazil, 2012.

Type of support	Level of support	OR	p	95%CI	
				Minimum	Maximum
Institutional (n = 14,306)	Low	1	–	–	–
	Medium	1.96	0.01	1.86	2.06
	High	3.77	0.001	3.52	4.04
Matrix (n = 14,489)	Low	1	–	–	–
	Medium	1.79	0.01	1.696	1.892
	High	3.29	0.001	3.087	3.514

Reference category for the dependent variable: regular certification.

Table 4 shows the detailed association between the presence of institutional support types and team certification, with the fair certification being the reference for the dependent variable. Very good or good certification was positively associated with self-assessment activities (OR = 1.95), permanent education (OR = 1.43), shared evaluation (OR = 1.40), and supervision and evaluation of indicators (OR = 1.37). On the other hand, receiving support for the discussion of primary healthcare teams’ work processes reduces their chances of having very good or good certification (OR = 0,62) (Table 4).

**Table 4:** Association between the presence of institutional support types and team certification (PMAQ-AB). Brazil, 2012. (N = 14,306)

Type of support	Support	OR	p	95%CI	
				Minimum	Maximum
Discussion on work processes	No	1	0.001	0.56	0.69
	Yes	0.62			
Self-assessment	No	1	0.001	1.78	2.15
	Yes	1.95			
Supervision and evaluation of indicators	No	1	0.001	1.24	1.52
	Yes	1.37			
Shared evaluation	No	1	0.001	1.28	1.53
	Yes	1.40			
Permanent education	No	1	0.01	1.32	1.55
	Yes	1.43			

Source: Ministry of Health.^d^

Reference category for the dependent variable: regular certification

Table 5 shows the association between the presence of MS types and team certification, with the fair certification being the reference for the dependent variable. Very good or good certification is positively associated with permanent education activities (OR = 1.50), interventions in the territory (OR = 1.30), and discussion in work processes (OR = 1.23). It should be pointed out that the conduction of MS for joint construction, clinical appointments, and shared clinical actions do not interfere much in the chance for teams to obtain good certification (OR = 1.15, 1.12, and 1.10, respectively) (Table 5).

**Table 5:** Association between the presence of institutional support types and team certification. PMAQ-AB Brazil, 2012. (N = 14,489)

Type of support	Support	OR	p	95%CI	
				Minimum	Maximum
Clinical appointments	No	1	0.014	1.02	1.18
	Yes	1.10			
Shared clinical actions	No	1	0.023	1.02	1.23
	Yes	1.12			
Joint construction	No	1	0.002	1.05	1.25
	Yes	1.15			
Permanent education	No	1	0.001	1.38	1.63
	Yes	1.50			
Discussions on work processes	No	1	0.001	1.12	1.35
	Yes	1.23			
Interventions in the territory	No	1	0.001	1.19	1.42
	Yes	1.30			

Source: Ministry of Health.^d^

Reference category for the dependent variable: regular certification

## DISCUSSION

This study showed that the IS and MS activities were expressive among BHC teams who took part in PMAQ-AB in 2012. Most teams performed more than four IS and MS activities (82.5% and 72.2%, respectively. The level of support teams receive – whether it is institutional or matrix – was associated with PMAQ-AB’s certification result. That is, the more supporting activities are incorporated, the better the result that is obtained in the certification of teams is.

The strong association between the levels of the received support and the certification of teams corroborate the hypothesis that the execution of organizational activities that foster the dissemination of knowledge and critical thinking improves BHC quality and management.^3^ The teams classified with high IS and MS levels had 277.0% and 229.0%, respectively, of chances for obtaining better certification ratings concerning PMAQ.

IS, by its programmed activities, has been found to be present, as virtually all kinds of actions were found to have high frequency percentages, with the exception of team planning and organization and workshop with a specific objective. That presence may be indicative of a shift towards chances in management practices, thus offering support for a qualified evaluation of the service.^11^

The action from institutional sponsors involves their inclusion in collective spaces, in a way to enable teams to self-assess and deeply analyze the aspects which interfere in the work of professionals. That dimension was observed in this study, as the performance of self-assessment by the teams was found to be significant (around 81.0% of evaluated teams pointed out that kind of support) and to be strongly associated with the certification of teams. Self-assessment implies reflection from managers and teams to identify problems and solutions, planning ways to deal with challenges in primary healthcare management. Besides that, self-assessment tends to result in shared modes of action that lead all parties involved to be responsible for situations identified as problems.^5^

In turn, in the dimension regarding specific qualifications of primary healthcare professionals, IS strategies regarding permanent education may contribute to professional development and enhance work processes.^5^ As mentioned by Oliveira et al^14^ in a study that was conducted in Bahia, Northeastern Brazil, IS considers permanent education as a structuring guideline and a management tool that ultimately allows management teams to fully understand the complex everyday events in health care units. That consideration may be observed in the positive association that was found between the conduction of permanent education and certification activities by teams, as teams had 43.0% chances of obtaining very good or good certification ratings with those activities.

The positive associations between the following IS types – shared evaluation and supervision and evaluation of indicators – and team certification were also significant. Those results suggest that IS activities are strategies under constant construction and serve to guide shared management practices.^3^

On the other hand, although discussion on work processes supporting activity, which is considered to be important for the supervision and construction of change processes for management practices, is more frequent in basic care teams, it has not yielded a positive association with the certification of teams. That fact relates to the possibility from institutional sponsors that still face troubles to deal with the entanglement of forces supporting the conflicts, and, at the same time, to have conditions to implement analysis processes with teams. The IS proposal is based on a reflection on the everyday factors in work processes and their implications.^1^ Sponsors and BHC teams often face obstacles due to the fragmented modes of action which are still present in health care services. Also, in that IS activity, the different concepts and practices from team members can also be found to be frequently conflicting or contradicting. As presented in a case study that was conducted in a BHC unit in Porto Alegre,^16^ RS, Southern Brazil, team members were noted to have different motivations to work, as well as different guiding principles, expectations for change, and ways to deal with the shared management. That IS activity may be the one requiring the greatest efforts for shared management practices to advance in BHC.

In regards to MS, over 70.0% of teams stated they performed activities such as clinical appointments, discussion on clinical cases, shared clinical actions, and visits with professionals. That reality was expected, as they were activities strongly motivated in BHC and health care environments. The health care area was the first one to discuss MS in Brazil.^7^ ^,^ ^10^

However, this study showed that only 22.0% of the teams mentioned they have received support to reorganize their work processes, and that was the least frequent activity. Those data deserve attention, once the organization of work processes is fundamental for teams to achieve effectiveness in health care services. Discussing work processes becomes even more important in the case of teams working with MS, in which the challenge and complexity concerning working with other professionals in the BHC team, either as reference or by incorporating them in the teams, requires the establishment of a horizontal relationship among professionals. To that end, several cross hierarchy lines have to be implemented, and the relationship between reference, supporting, and specialist teams’ needs to be ordered based on dialog-oriented procedures.^3^ ^,^ ^6^

An effective MS process implies changes in how health care services and systems work and are organized. Such dynamics guides the relationship among the hierarchical levels in the system, and it makes the communication and integration among BHC teams and specialists easier.^3^ ^,^ ^8^ That is, the high number of teams, as per PMAQ-AB, which receive MS should be more effectively based on the re-structuring of work processes. Therefore, the MS consolidation as a catalyst for changes in the care model is still in progress and needs to be enhanced. However, one should acknowledge that the shift in the work logics that was proposed by MS does not occur automatically, and it must be specifically dealt with, based on the implementation of spaces destined for reflection and critics on work processes themselves.^9^

The identified MS activities that lead to better certification are, in order of importance, the following: permanent education, interventions in the territory, joint construction of therapeutic processes, and discussion in work processes, and they are followed, to a lesser extent, by shared clinical actions and clinical appointments. As expected, the MS activities that interfere the most in better quality certification are the ones concerning interaction processes, namely the permanent education actions. Despite activity discussion on work processes being less frequent in BHC teams, we have observed how much it influenced the certification of teams, thus showing its importance in compliance with the literature.^3^ ^,^ ^8^ ^,^ ^9^

The seeming paradoxical relationship found in this study concerning activity discussion on work processes must be further explored in future research, including in other PMAQ-AB cycles, as it was the most frequent activity for IS, but it was not positively correlated with the certification of teams. On the other hand, for MS it was the least frequent activity; however, it was associated with certification. That scenario reinforces the importance of the role of IS and MS for the discussion on work practices, as this is one of the fundamental aspects for the implementation of measures which improve the health care quality.

Our study has limitations. The first PMAQ-AB cycle limited the participation to 50.0% of the teams, which may have added a selection bias, as it would lead the most structured teams in each municipality to take part – consequently, those would achieve better certification. However, IS and MS account for only 0.1% of total certification.^e^ The aforementioned study may also have had an information bias, as IS is a strategy that is fostered by the Ministry of Health as an action that can improve BHC quality in the teams. Those issues may be better clarified when the data from the next PMAQ-AB cycle are analyzed, as virtually all teams had access to it.

The results in this study show the spread of supporting strategies, whether they are institutional or matrix, and they have gained ground in the everyday work of health care teams. Those findings may help managers get organized to better qualify the provided support that can decisively reflect improvements in the quality and access to primary healthcare services in Brazil.

## Footnotes

a Ministério da Saúde. Caderno de referência para o processo de formação de profissionais do Apoio Institucional Integrado do Ministério da Saúde: QualiSUS-Rede. Brasília (DF); 2011. (Série F. Comunicação e Educação em Saúde).

b Campos GWS. Efeito Paidéia e humanização em saúde: cogestão e não violência como postura existencial e política. Brasília (DF): Ministério da Saúde, Secretaria de Atenção Básica; 2010. (Caderno Humaniza SUS, 2. Atenção Básica).

c Pereira Júnior N. O apoio institucional no SUS: os dilemas da integração interfederativa e da cogestão [dissertation]. Campinas (SP): Universidade Estadual de Campinas; 2013.

d Ministério da Saúde, Secretaria de Atenção à Saúde, Departamento de Atenção Básica. Saúde mais perto de você: acesso e qualidade - Programa Nacional de Melhoria do Acesso e da Qualidade da Atenção Básica (PMAQ): manual instrutivo. Brasília (DF); 2012. (Série A. Normas e Manuais Técnicos).

e Ministério da Saúde, Secretaria de Atenção à Saúde, Departamento de Atenção Básica. Nota metodológica da certificação das Equipes de Atenção Básica participantes do Programa de Melhoria do Acesso e da Qualidade na Atenção Básica. Brasília (DF); 2013 [cited 2015 Feb 8]. p.37. Available from: http://189.28.128.100/dab/docs/portaldab/documentos/nota_metodologica_pmaq.pdf
