# Supplementary material for: Institutional and matrix support and its relationship with primary healthcare
Source: Rev Saude Publica. 2015 Sep 10;49:54. doi: 10.1590/S0034-8910.2015049005519 (PMC4544363; doi:10.1590/S0034-8910.2015049005519)
Supplement: Translation

# Translation: Apoio institucional e matricial e sua relação com o cuidado na atenção básica à saúde

**Authors:** Alaneir de Fátima dos Santos, Antônio Thomaz Gonzaga da Matta Machado, Clarice Magalhães Rodrigues dos Reis, Daisy Maria Xavier Abreu, Lucas Henrique Lobato de Araújo, Simone Cristina Rodrigues, Ângela Maria de Lourdes Dayrell de Lima, Alzira de Oliveira Jorge, Délcio Fonseca

**Affiliations:** Núcleo de Educação em Saúde Coletiva. Faculdade de Medicina. Universidade Federal de Minas Gerais. Belo Horizonte, MG, Brasil

**Keywords:** Atenção Primária à Saúde, organização & administração, Apoio ao Planejamento em Saúde, Estudos Transversais

## Abstract

**OBJETIVO:**

Analisar se o grau de apoio institucional e matricial está associado à melhor certificação das equipes da atenção básica à saúde.

**MÉTODOS:**

Neste estudo transversal analisamos dois tipos de apoio na atenção básica, sendo que 14.489 equipes receberam apoio institucional e 14.306 equipes receberam apoio matricial. Foram aplicados modelos de regressão logística. No de apoio institucional, a variável independente foi grau de apoio (calculado pelo somatório de atividades de apoio das duas modalidades), enquanto no de apoio matricial, as variáveis independentes foram as atividades de apoio. A análise multivariada considerou variáveis que apresentaram p < 0,20. O ajuste do modelo foi realizado pelo teste de Hosmer-Lemeshow.

**RESULTADOS:**

As equipes contavam com atividades de apoio institucional e matricial em 84,0% e 85,0%, respectivamente, sendo que 55,0% realizavam entre seis e oito atividades. Para o apoio institucional, observamos chances de 1,96 e 3,77 de possuir certificação ótima ou boa quando as equipes possuíam médio e alto grau de apoio, respectivamente. Para o apoio matricial, as chances de possuir certificação ótima ou boa foram de 1,79 e 3,29, respectivamente. Quanto à associação entre atividades de apoio institucional e a certificação, a certificação ótima ou boa associou-se positivamente com autoavaliação (OR = 1,95), educação permanente (OR = 1,43), avaliação compartilhada (OR = 1,40) e monitoramento e avaliação de indicadores (OR = 1,37). Quanto ao apoio matricial, a certificação ótima ou boa associou-se positivamente com educação permanente (OR = 1,50), intervenções no território (OR = 1,30) e discussão nos processos de trabalho (OR = 1,23).

**CONCLUSÕES:**

No Brasil, as atividades de apoio estão sendo incorporadas na atenção básica, existindo associação entre o grau de apoio tanto matricial quanto institucional e o resultado da certificação.

## INTRODUÇÃO

A construção de um modelo assistencial que tenha a atenção primária à saúde como coordenadora do cuidado na rede de atenção básica à saúde impõe desafios que, no Brasil, são enfrentados com propostas que procuram concretizar o princípio da integralidade no Sistema Único de Saúde (SUS). Para confrontar as tradicionais formas de funcionamento dos serviços de saúde, que mantêm processos de trabalho fragmentados, centrados em procedimentos e com foco de atuação no modelo biomédico hegemônico, surgem, por parte do SUS, as propostas de apoio institucional (AI) e apoio matricial (AM).^2^

O AI está pautado na cogestão e tem como objetivo desenvolver e aprimorar estratégias de supervisão e avaliação dos serviços de saúde.^a^ Tal proposta pressupõe um espaço coletivo de articulação entre gestão e cuidado, incentivando a participação dos sujeitos em coletivos organizados visando a produzir bens ou serviços e promover a educação permanente e a gestão compartilhada da instituição e de seus processos de trabalho.^5^

A produção sistemática de documentos oficiais que abordam a estratégia do AI ocorreu a partir de 2003, influenciada pelo Apoio Paidéia, desenvolvida por Campos.^5^ Nessa concepção, gestão e práticas de saúde aproximam-se na tentativa de desenvolver uma gestão democrática – a cogestão.^b^ ^,^ ^c^

Já o AM é uma estratégia de organização do trabalho em que a atuação dos profissionais de saúde deve ser feita em equipes multiprofissionais.^2^ Assim, o apoio de profissionais capacitados às equipes de atenção básica à saúde (ABS) pode assegurar o cuidado integral, propondo responsabilidade mútua com os usuários do serviço.^2^ O AM investe em mudanças da estrutura gerencial e assistencial dos serviços de saúde, de modo a criar uma organização que estimule o compromisso das equipes com a produção de saúde, sem cobrar-lhes onisciência ou onipotência, e permitindo-lhes, ao mesmo tempo, sua própria realização pessoal e profissional. O AM refere-se, assim, a um novo arranjo que estimule, cotidianamente, a produção de novos padrões de inter-relação entre equipe e usuários e que amplie o compromisso dos profissionais com a produção de saúde, quebrando obstáculos organizacionais à comunicação.^4^

O dispositivo AM pode problematizar as práticas e sinalizar os caminhos para a construção de outro modelo de assistência.^12^ As primeiras experiências de AM ocorreram em Campinas, SP, a partir de 1989, em dois serviços de saúde mental, que passaram a funcionar com equipes multiprofissionais. Posteriormente, em Betim, MG, se expandiu e, em 2003, o Ministério da Saúde o adotou na estruturação da saúde mental.^4^ Em 2009, o Ministério da Saúde concretizou o AM com a institucionalização dos Núcleos de Apoio à Saúde da Família na estrutura organizacional da ABS no SUS. Alguns estudos mostram os benefícios advindos do AM, particularmente em saúde mental.^9^ ^,^ ^10^ ^,^ ^12^ ^,^ ^13^ ^,^ ^15^

Diante da expectativa de que esses mecanismos avancem, torna-se necessário caracterizar a situação do AI e AM na ABS. Para isso, a avaliação externa do Programa de Melhoria do Acesso e Qualidade da Atenção Básica (PMAQ-AB)^d^ oferece informações sobre os processos de trabalho na ABS, contemplando dimensões do AI e AM em todo o território nacional.

O objetivo deste estudo foi analisar se o grau de apoio institucional e matricial está associado a melhor certificação das equipes da atenção básica à saúde.

## MÉTODOS

Estudo transversal com base no banco de dados da avaliação externa do PMAQ-AB – primeiro ciclo. Foram realizadas, em 2012, entrevistas com 17.055 profissionais de nível superior da equipe de ABS, distribuídos em todos os estados brasileiros. Foram analisados os dados relativos às equipes que receberam alguma atividade de AI e AM, correspondendo a 14.306 (84,0%) e 14.489 (85,0%), respectivamente.

Foram utilizadas as variáveis do questionário II do PMAQ-AB, que identificam as atividades de AI e AM oferecidas às equipes avaliadas. Os tipos de AI e AM considerados pelo PMAQ-AB estão detalhados na Tabela 1. Também consideramos o resultado obtido pelas equipes na certificação adotada pelo PMAQ-AB: ótimo, bom e regular.

**Tabela 1:** Tipos de apoio institucional e matricial analisados pelo Programa Nacional de Melhoria do Acesso e da Qualidade da Atenção Básica (PMAQ-AB). Brasil, 2012.

Apoio institucional	n	%	Apoio matricial	n	%
Discussão sobre processo de trabalho das equipes de atenção básica	12.893	90,1	Consultas clínicas	12.825	88,5
Apoio à autoavaliação	11.558	80,8	Discussão de casos clínicos	10.662	73,6
Monitoramento e avaliação de indicadores	11.773	82,3	Ações clínicas compartilhadas	10.547	72,8
Planejamento e organização da equipe	4.723	33,0	Construção conjunta de processos terapêuticos	7.921	54,7
Avaliação compartilhada	9.760	68,4	Educação permanente	8.936	61,7
Oficina com objetivo específico	6.955	48,6	Discussões processo de trabalho com a equipe	3.308	22,8
Educação permanente	9.068	63,4	Intervenções no território com a equipe	9.400	64,9
Reuniões com a equipe	10.510	73,5	Visitas com os profissionais	10.507	72,5
Outros apoios	441	2,5			
Total	14.306			14.489	

Fonte: Ministério da Saúde.^d^

Inicialmente, foram verificadas as atividades de apoio recebido pelas equipes por meio de frequências absolutas e relativas. Criou-se a variável grau de apoio, referente à quantidade de apoio recebida pelas equipes. Essa variável foi calculada pelo somatório de atividades de AM e AI recebidas pela equipe. Posteriormente, o grau de AI e AM foi categorizado de acordo com a quantidade de atividades de apoio recebidas, sendo: apoio baixo (uma a três atividades), apoio médio (quatro a seis atividades) e apoio alto (sete ou oito atividades).

Para verificar associações entre o apoio recebido pelas equipes e a qualidade da atenção foi realizada a análise de regressão logística binária múltipla, tendo como variável dependente o resultado da certificação das equipes. Essa variável foi categorizada em certificação “ótimo ou boa” e “regular ou insuficiente” e foram aplicados dois modelos de regressão logística múltipla. No primeiro modelo, a variável independente foi o grau de apoio das equipes para AI e AM, enquanto no segundo, a variável independente foi “atividades de AI e AM identificadas no PMAQ-AB”. A análise multivariada considerou aquelas variáveis que, na análise univariada, apresentaram p < 0,20. Foram adotados como nível de significância estatística p < 0,05 e intervalo de 95% de confiança (IC95%), com cálculo da respectiva *odds ratio* (OR) para indicar a magnitude das associações. As estimativas de OR e IC95% de cada variável foram ajustadas para o efeito das demais variáveis estudadas no modelo. O ajuste do modelo múltiplo final foi feito por meio do teste de Hosmer-Lemeshow.

## RESULTADOS

A Tabela 1 detalha a frequência relativa e absoluta dos tipos de apoio institucional e matricial analisados pelo PMAQ-AB. As principais atividades de AI foram: discussão do processo de trabalho das equipes de atenção básica (90,1%), monitoramento e avaliação dos indicadores (82,3%) e apoio à autoavaliação (80,8%), enquanto planejamento e organização da equipe apresentou a menor frequência (33,0%) (Tabela 1). Em relação ao AM, as atividades mais presentes nas equipes de ABS foram: consultas clínicas (88,5%), discussão de casos clínicos (73,6%), ações clínicas compartilhadas (72,8%) e visita com os profissionais (72,5%), enquanto a discussão sobre o processo de trabalho com a equipe foi a atividade de AM menos envolvida pelas equipes (22,8%).

Observamos que a maior parcela das equipes contava com mais de um tipo de apoio. Apenas 3,6% e 8,8% delas receberam uma única atividade de AI e AM, respectivamente. Em relação ao AI, 17,1% das ESF possuíam apoio baixo, 47,7% apoio médio e 34,8%, alto grau de apoio. Quanto ao AM, 25,9% das equipes possuíam baixo apoio e 38,7% e 35,5% possuíam médio e alto grau de apoio, respectivamente (Tabela 2).

**Tabela 2:** Distribuição do percentual de tipos de apoio institucional e matricial (PMAQ-AB). Brasil, 2012.

Quantidade de apoio	Grau de apoio	Apoio institucional		Apoio matricial	

		n	%	n	%
0		62	0,4	–	–
1	Baixo	522	3,6	1.271	8,8
2		758	5,3	1.113	7,7
3		1.179	8,2	1.362	9,4
4	Médio	1.585	11,1	1.483	10,2
5		2.298	16,1	1.853	12,8
6		2.932	20,5	2.272	15,7
7	Alto	3.487	24,4	3.386	23,4
8		1.483	10,4	1.749	12,1
Total		14.306	100	14.489	100

Fonte: Ministério da Saúde.^d^

Para a análise de associação entre grau de apoio e certificação, considerou-se como categoria de referência da variável dependente a certificação regular e a resposta ótimo ou boa. Observou-se aumento da chance de possuir certificação ótima ou boa de acordo com o grau de apoio recebido. Para o AI, verificou-se chance de 1,96 e 3,77 de possuir certificação ótima ou boa quando as equipes possuíam médio e alto grau de apoio, respectivamente. Para o AM, as chances de possuir certificação ótima ou boa foram de 1,79 e 3,29, respectivamente. Ou seja, receber um grau de apoio alto, seja matricial ou institucional, eleva a chance de uma equipe receber melhor certificação (Tabela 3).

**Tabela 3:** Associação entre o grau de apoio institucional e matricial e a certificação da equipe (PMAQ-AB). Brasil, 2012.

Tipo de apoio	Grau de apoio	OR	p	IC95%	
				Mínimo	Máximo
Institucional (n = 14.306)	Baixo	1	–	–	–
	Médio	1,96	0,01	1,86	2,06
	Alto	3,77	0,001	3,52	4,04
Matricial (n = 14.489)	Baixo	1	–	–	–
	Médio	1,79	0,01	1,696	1,892
	Alto	3,29	0,001	3,087	3,514

Categoria de referência da variável dependente: certificação regular.

A Tabela 4 detalha associação entre a presença dos tipos de AI e a certificação das equipes, tendo a certificação regular como referência da variável dependente. Verifica-se que a certificação ótima ou boa está associada positivamente com atividades de autoavaliação (OR = 1,95), educação permanente (OR = 1,43), avaliação compartilhada (OR = 1,40) e monitoramento e avaliação de indicadores (OR = 1,37). Por outro lado, receber apoio para discussão sobre o processo de trabalho da equipe de atenção básica reduz a chance de possuir certificação ótima ou boa (OR = 0,62) (Tabela 4).

**Tabela 4:** Associação entre a presença dos tipos de apoio institucional e certificação das equipes (PMAQ-AB). Brasil, 2012. (N = 14.306)

Tipo de apoio	Apoio	OR	p	IC95%	
				Mínimo	Máximo
Discussão sobre o processo de trabalho	Não	1	0,001	0,56	0,69
	Sim	0,62			
Autoavaliação	Não	1	0,001	1,78	2,15
	Sim	1,95			
Monitoramento e avaliação de indicadores	Não	1	0,001	1,24	1,52
	Sim	1,37			
Avaliação compartilhada	Não	1	0,001	1,28	1,53
	Sim	1,40			
Educação permanente	Não	1	0,01	1,32	1,55
	Sim	1,43			

Fonte: Ministério da Saúde.^d^

Categoria de referência da variável dependente: certificação regular.

A Tabela 5 descreve a associação entre a presença dos tipos de AM e a certificação das equipes, tendo como referência a certificação regular. Observa-se que a certificação ótima ou boa está associada positivamente com atividades de educação permanente (OR = 1,50), intervenções no território (OR = 1,30) e discussão nos processos de trabalho (OR = 1,23). Ressalta-se que a realização de AM para construção conjunta, consultas clínicas e ações clínicas compartilhadas interferem pouco na chance de obter uma boa certificação (OR = 1,15, 1,12 e 1,10, respectivamente) (Tabela 5).

**Tabela 5:** Associação entre a presença dos tipos de apoio matricial e a certificação das equipes. PMAQ-AB Brasil, 2012. (N = 14.489)

Tipo de apoio	Apoio	OR	p	IC95%	
				Mínimo	Máximo
Consultas clínicas	Não	1	0,014	1,02	1,18
	Sim	1,10			
Ações clínicas compartilhadas	Não	1	0,023	1,02	1,23
	Sim	1,12			
Construção conjunta	Não	1	0,002	1,05	1,25
	Sim	1,15			
Educação permanente	Não	1	0,001	1,38	1,63
	Sim	1,50			
Discussões processo de trabalho	Não	1	0,001	1,12	1,35
	Sim	1,23			
Intervenções no território	Não	1	0,001	1,19	1,42
	Sim	1,30			

Fonte: Ministério da Saúde.^d^

Categoria de referência da variável dependente: certificação regular.

## DISCUSSÃO

Este estudo mostrou que as atividades de AI e AM foram expressivas dentre as equipes de ABS que participaram do PMAQ-AB em 2012. A maioria das equipes desenvolviam mais de quatro atividades de AI e AM (82,5% e 72,2%, respectivamente). O grau de apoio que a equipe recebe – seja institucional ou matricial – associou-se ao resultado da certificação do PMAQ-AB. Ou seja, quanto maior a incorporação das atividades de apoio, maior o resultado obtido na certificação das equipes.

A forte associação entre os graus de apoio recebidos e a certificação das equipes corrobora a hipótese de que a execução de atividades organizacionais, fomentando a circulação de saberes e capacidade crítica dos grupos, melhora a qualidade e a gestão da ABS.^3^ As equipes classificadas com grau de AI e AM alto possuíam, respectivamente, 277,0% e 229,0% de chance de obter melhor certificação no âmbito do PMAQ.

O AI, por meio das atividades programadas, tem se mostrado presente, pois praticamente todos os tipos de ações apresentaram elevado percentual de frequência, exceto planejamento e organização da equipe e oficina com objetivo específico. Essa presença pode ser indicativa de um movimento de mudanças nas práticas de gestão, oferecendo subsídios para uma avaliação qualificada do serviço.^11^

A atuação do apoiador institucional envolve sua inserção em espaços coletivos, de maneira a permitir às equipes o exercício de autoanálise e uma análise aprofundada dos aspectos que interferem na atuação dos profissionais. Essa dimensão foi observada neste estudo, pois a realização de autoavaliação pelas equipes mostrou-se expressiva (cerca de 81,0% das equipes avaliadas apontaram esse tipo de apoio) e apresentou forte associação com a certificação das equipes. A autoavaliação pressupõe uma reflexão dos gestores e equipes para a identificação de problemas e soluções, planejando modos de enfrentar os desafios apresentados na gestão do cuidado na atenção básica. Além disso, a autoavaliação tende a provocar modos de ação compartilhados que levem a uma responsabilização do coletivo pela mudança da situação identificada como problema.^5^

Já na dimensão da formação específica do profissional para atuar na atenção básica, as estratégias de AI para educação permanente podem contribuir com o desenvolvimento profissional e qualificar o processo de trabalho.^5^ Como apontado por Oliveira et al^14^ em estudo realizado na Bahia, o AI considera a educação permanente como uma diretriz estruturante e uma ferramenta de gestão que, em última instância, permite a apropriação, por parte da equipe de gestão, do complexo cotidiano presente nas unidades de saúde. Essa consideração pode ser observada na associação positiva encontrada entre a realização de atividades de educação permanente e certificação das equipes, já que com essas atividades as equipes tiveram 43,0% de chance de obterem certificação ótima ou boa.

Foram também significativas as associações positivas entre os tipos de AI – avaliação compartilhada e monitoramento e avaliação de indicadores – e a certificação das equipes (1,40 e 1,37, respectivamente). Esses resultados sugerem que as atividades de AI se apresentam como estratégias em constante construção, balizadoras das práticas de gestão compartilhada.^3^

Por outro lado, apesar de a atividade de apoio discussão sobre o processo de trabalho, considerada importante para acompanhamento e construção dos processos de mudança nas práticas gerenciais, ser mais frequente nas equipes de AB não resultou em associação positiva com a certificação das equipes. Essa questão remete à possibilidade de o apoiador institucional ainda enfrentar dificuldades para lidar com o emaranhado de forças que sustentam os conflitos e, ao mesmo tempo, ter condições de disparar processos de análise junto às equipes. A proposta de AI é pautada em uma reflexão do cotidiano dos processos de trabalho e de suas implicações^1^ e, muitas vezes, o apoiador e as equipes de ABS se deparam com entraves face aos modos de ação fragmentados ainda presentes nos serviços de saúde. Ainda, nessa atividade do AI, podem ser evidenciadas as diferentes concepções e práticas frequentemente conflituosas ou contraditórias dos membros da equipe. Conforme foi apresentado em estudo de caso realizado em uma unidade de ABS em Porto Alegre,^16^ foram percebidas diferentes motivações para o trabalho entre os integrantes da equipe, com diversos princípios orientadores, expectativas de mudança e formas de lidar com a gestão compartilhada. Essa atividade do AI é talvez a que mais demanda esforço para que se avance nas práticas de gestão compartilhada na ABS.

Em relação ao AM, mais de 70,0% das equipes afirmaram realizar atividades como consultas clínicas, discussão de casos clínicos, ações clínicas compartilhadas e visitas com os profissionais. Essa realidade era esperada por serem atividades fortemente incentivadas na interface ABS e saúde mental, que foi pioneira na discussão de AM no Brasil.^7^ ^,^ ^10^

No entanto, o presente estudo mostrou que somente 22,0% das equipes referem receber apoio para a reorganização do processo de trabalho, constituindo-se a atividade menos frequente. Esses dados merecem atenção, uma vez que a organização do processo de trabalho é fundamental para a efetividade do cuidado em saúde prestado pela equipe. Discutir o processo de trabalho torna-se ainda mais importante no caso de equipes que trabalham com AM, onde o desafio e a complexidade de trabalhar com outros profissionais na equipe de ABS, seja como referência ou incorporando-os nas equipes, demanda estabelecer uma relação horizontal entre os profissionais. Para isso, é necessário construir várias linhas de transversalidade e ordenar essa relação entre equipe de referência, apoios e especialistas com base em procedimentos dialógicos.^3^ ^,^ ^6^

Um processo de AM efetivo pressupõe transformações no modo como se organizam e funcionam serviços e sistemas de saúde. Essa dinâmica ordena a relação entre os níveis hierárquicos do sistema e facilita a comunicação e integração de equipes de ABS e especialistas.^3^ ^,^ ^8^ Ou seja, a magnitude encontrada nos dados do PMAQ-AB de equipes que recebem AM deveria estar, de forma mais efetiva, ancorada em reestruturação de processos de trabalho. Portanto, a consolidação do AM como indutor de mudança no modelo assistencial ainda está em andamento e precisa ser aprimorada. Deve-se reconhecer, no entanto, que a mudança da lógica de trabalho proposta pelo AM não ocorre automaticamente e deve ser especificamente trabalhada, instalando espaços destinados à reflexão e análise crítica sobre o próprio processo de trabalho.^9^

As atividades de AM identificadas que levam a melhor certificação são, em ordem de magnitude: educação permanente, intervenções no território, construção conjunta de processos terapêuticos e discussão nos processos de trabalho, seguidas em menor grau por ações clínicas compartilhadas e consultas clínicas. Como esperado, as atividades de AM que mais interferem para uma melhor certificação da qualidade são aquelas referentes a processos de interação, com destaque para as ações de educação permanente. Apesar de a atividade discussão de processo de trabalho ser menos frequente nas equipes de ABS, observamos o quanto ela impactou na certificação das equipes, mostrando sua importância em conformidade com a literatura.^3^ ^,^ ^8^ ^,^ ^9^

A aparente relação paradoxal encontrada neste estudo quanto à atividade discussões sobre processo de trabalho deve ser mais explorada em próximas pesquisas, inclusive em outros ciclos do PMAQ-AB, pois, se para AI foi a mais frequente atividade realizada, não apresentou relação positiva com a certificação das equipes. Por outro lado, para AM foi a atividade menos frequente, porém com associação com a certificação. Esse cenário reforça a importância do papel do AI e AM para a discussão das práticas de trabalho, visto que esse é um dos aspectos fundamentais para implementação de medidas que melhorem a qualidade da atenção à saúde.

O presente estudo apresenta limitações. O primeiro ciclo do PMAQ-AB limitou a adesão a 50,0% das equipes, o que pode ter acrescentado um viés de seleção, pois levaria à adesão das equipes mais estruturadas de cada município que, por conseguinte, podem apresentar melhor certificação. No entanto, o peso de AI e AM representa apenas 0,1% no total da certificação.^e^ Este citado estudo pode também ter apresentado viés de informação, pois o AI é uma estratégia incentivada pelo Ministério da Saúde como ação que pode melhorar a qualidade da ABS nas equipes. Essas questões podem ser mais esclarecidas quando os dados do próximo ciclo do PMAQ-AB forem analisados, visto que neles a adesão das equipes foi praticamente universal.

Os resultados desta pesquisa mostram a capilaridade das estratégias de apoio, seja institucional ou matricial, que ganharam espaço no cotidiano do trabalho das equipes de saúde. Estes achados podem contribuir para os gestores se organizarem para qualificar melhor os apoios ofertados, incidindo de forma decisiva na melhoria da qualidade e acesso da atenção básica à saúde no Brasil.

## Footnotes

a Ministério da Saúde. Caderno de referência para o processo de formação de profissionais do Apoio Institucional Integrado do Ministério da Saúde: QualiSUS-Rede. Brasília (DF); 2011. (Série F. Comunicação e Educação em Saúde).

b Campos GWS. Efeito Paidéia e humanização em saúde: cogestão e não violência como postura existencial e política. Brasília (DF): Ministério da Saúde, Secretaria de Atenção Básica; 2010. (Caderno Humaniza SUS, 2. Atenção Básica).

c Pereira Júnior N. O apoio institucional no SUS: os dilemas da integração interfederativa e da cogestão [dissertação]. Campinas (SP): Universidade Estadual de Campinas; 2013.

d Ministério da Saúde, Secretaria de Atenção à Saúde, Departamento de Atenção Básica. Saúde mais perto de você: acesso e qualidade - Programa Nacional de Melhoria do Acesso e da Qualidade da Atenção Básica (PMAQ): manual instrutivo. Brasília (DF); 2012. (Série A. Normas e Manuais Técnicos).

e Ministério da Saúde, Secretaria de Atenção à Saúde, Departamento de Atenção Básica. Nota metodológica da certificação das Equipes de Atenção Básica participantes do Programa de Melhoria do Acesso e da Qualidade na Atenção Básica. Brasília (DF); 2013 [citado 2015 fev 8]. p.37. Disponível em: http://189.28.128.100/dab/docs/portaldab/documentos/nota_metodologica_pmaq.pdf
